# Combination Treatment with Depressor Anguli Oris Myectomy and Pedicled Buccal Fat Pad Flap for Sequelae of Facial Paralysis: Case Reports

**DOI:** 10.1055/a-2631-4203

**Published:** 2025-09-01

**Authors:** Ko Nakao, Eri Matoba, Hisashi Sakuma

**Affiliations:** 1 Department of Plastic and Reconstructive Surgery, Ichikawa General Hospital, Tokyo Dental College, Ichikawa, Japan

**Keywords:** selective myectomy, depressor anguli oris, depressor labii inferioris, pedicled buccal fat pad flap

## Abstract

Facial paralysis sequelae result in functional and cosmetic deficits. Myectomy for facial contractures has been reported, and recently, selective myectomy of the smile antagonists (depressor anguli oris [DAO]) for perioral synkinesis has gained attention. Although less invasive, this approach can lead to postoperative depressed deformities of the myectomy site. We report two cases of facial nerve paralysis. In one case, DAO myectomy was performed for synkinesis with the upper lip levator muscles. In the other, the DAO and depressor labii inferioris were myectomized for facial contractures centered on the lower lip. A pedicled buccal fat pad flap was elevated to cover the myectomy defect, preventing postoperative depressed deformity. One year postoperatively, no depressed deformities were observed; lower lip symmetry and oral commissure movement improved, achieving a natural smile. As the procedure involves the transfer of vascularized blood-rich buccal fat, the risk of postoperative induration and contracture is lower than that with fat injections.

## Introduction

Facial paralysis sequelae can significantly impair a patient's quality of life and may lead to devastating psychological consequences. Myectomy for facial contracture and blepharospasm has long been reported and is a useful technique. 1

Recently, some studies have suggested that the hypertonicity of the depressor anguli oris (DAO) may antagonize the oral commissure excursion, preventing the ability to smile. 2 3 This is considered to be mainly due to synkinesis of the zygomatic and buccal branches. 3 Therefore, treatments such as local injections of botulinum toxin into the DAO, selective myectomy, 4 and neurectomy target the hypertonicity of the DAO. 5 Among these techniques, myectomy is considered the most effective with the lowest recurrence rate, 5 although it often results in postoperative depressed deformities.

Previous techniques, such as superficial musculo-aponeurotic system (SMAS) grafting, dermal fat grafting, and fat injections, have been used for addressing these deformities. However, their non-vascularized nature leads to postoperative complications, including induration, scar contracture, and infection. 6 7

We observed that the pedicled buccal fat pad (PBFP) flap, commonly utilized in oral surgery, is capable of reaching the lower lip region. 8 9 This study describes the usefulness of myectomy with DAO and depressor labii inferioris (DLI) for managing synkinesis and hypertonicity. Additionally, it explores the potential of the PBFP flap to prevent postoperative depression deformities.

## Case

### Case 1

**Fig. 1:**
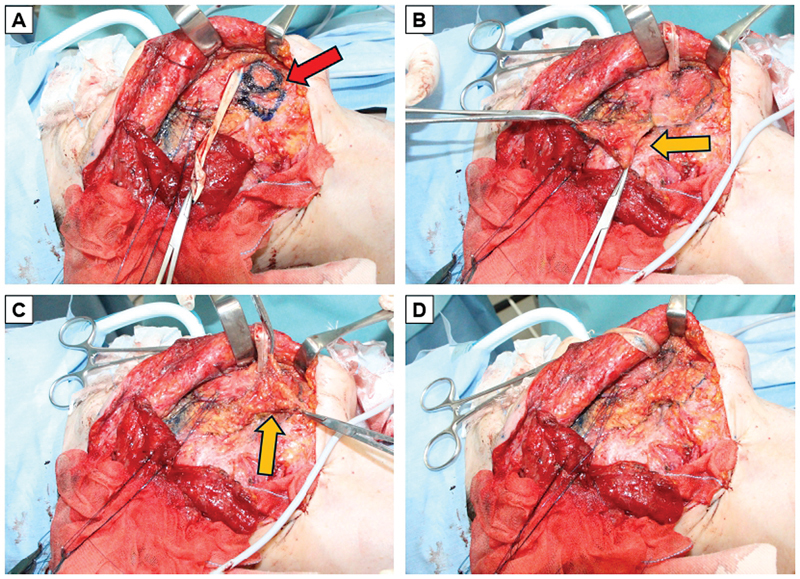
Intraoperative images of patient 1. ( **A** ) Selective myectomy for depressor anguli oris. ( **B, C** ) The PBFP flap was elevated to 25 × 10 mm (orange arrow). ( **D** ) The PBFP flap was transposed into the defect after myectomy. PBFP, pedicled buccal fat pad.

A 30-year-old male developed idiopathic right facial nerve palsy at age 22, resulting in deviation of the lower lip and limited oral commissure excursion despite initial treatment (House–Brackmann grade III). Since the DAO was palpated as a band during smiling, the patient was diagnosed with DAO hypertonicity, prompting a plan for DAO myectomy and augmentation with a PBFP flap. The procedure was performed under general anesthesia on an outpatient basis. A preauricular face-lift incision was made, elevating the facial flap in the SMAS plane, where the DAO was identified and excised to a length of 25 mm ( Fig. 1A ). To prevent postoperative edema, only the muscle was resected, preventing the surrounding tissues. 7 The SMAS was incised near the myectomy. The PBFP flap was elevated to 25 × 10 mm, including its capsule, as blood flow is capsule-dependent ( Fig. 1B, C ), 10 and transposed into the defect ( Fig. 1D ).

To address the deviation of the lower lip on the affected side during smiling, the double fascia graft technique described by Udagawa et al. 11 was employed to pull the lower lip toward the unaffected side and restore symmetry. Since the double fascia graft technique used the fascia lata, there was little postoperative sacrifice.

One year postoperatively, no depressed deformity was observed, and lower lip symmetry was significantly enhanced. The symmetry of the oral commissure was evaluated by the following method.

**Fig. 2:**
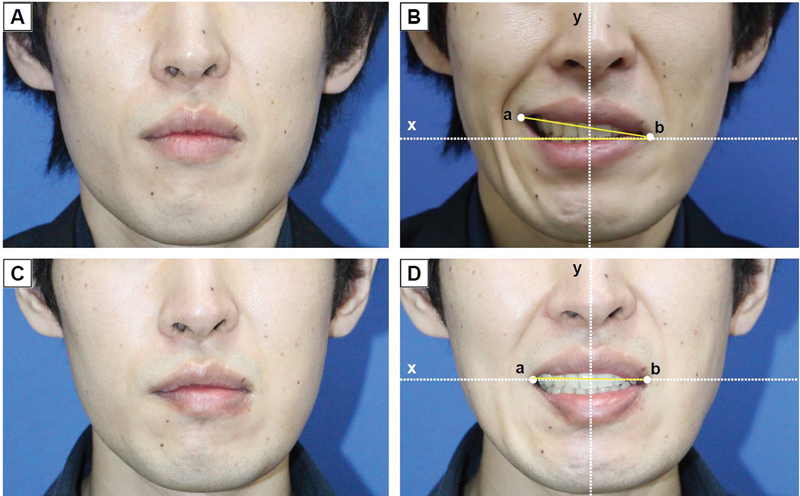
Preoperative and postoperative images of patient 1. ( **A** ) Preoperative image of the patient at rest. ( **B** ) Preoperative image showing perioral synkinesis with asymmetry in the corners of the mouth. (a) Oral commissure on the unaffected side; (b) oral commissure on the affected side; y, vertical bisector of the line connecting the centers of the pupils on both sides; x, line passing through the oral commissure on the affected side and perpendicular to the y-axis ∠abx was measured at 21 degrees. ( **C** ) Postoperative image of the patient at rest, taken 1 year after surgery. ( **D** ) Postoperative improvement in perioral synkinesis was observed 1 year after surgery. No depression deformity was evident in the perioral area, and symmetry of the mouth corners was restored. ∠abx was measured at 0 degrees.

First, the oral commissure on the unaffected and affected sides was defined as a and b, respectively. The vertical bisector of the line connecting the centers of the pupils on both sides was designated as the y-axis, while a line passing through the oral commissure on the affected side and perpendicular to the y-axis was designated as the x-axis. The symmetry of the mouth corners was demonstrated by comparing the angles of ∠abx pre- and postoperatively. There was an improvement in ∠abx from 21 degrees to 0 degrees pre- and postoperatively, resulting in a natural smile ( Fig. 2A–D ).

### Case 2

**Fig. 3:**
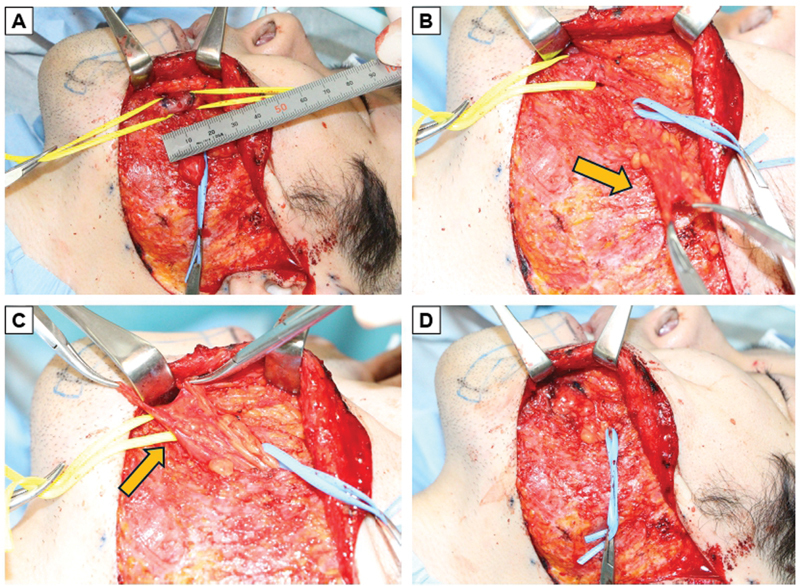
Intraoperative images of patient 2. ( **A** ) Selective myectomy for depressor anguli oris and depressor labii inferioris (red arrow). ( **B, C** ) PBFP flap was elevated to 40 mm × 10 mm (orange arrow). ( **D** ) PBFP flap was transposed into the defect after myectomy. PBFP, pedicled buccal fat pad.

**Fig. 4:**
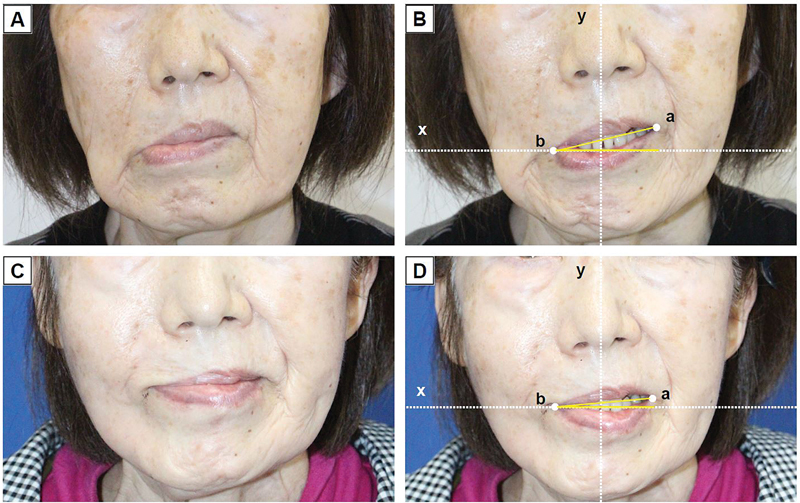
Preoperative and postoperative images of patient 2. ( **A** ) Preoperative image of the patient at rest. ( **B** ) Preoperative image showing muscle hypertonicity of the depressor anguli oris and depressor labii inferioris, with asymmetry in the corners of the mouth. ∠abx was measured at 13 degrees. ( **C** ) Postoperative image of the patient at rest, taken 1 year after surgery. ( **D** ) Postoperative relief of muscle hypertonicity and improvement in the drooping of the corners of the mouth. No depression deformity was observed in the perioral area. ∠abx was measured at 5 degrees.

A 72-year-old female developed right facial paresis at age 59 (House–Brackmann grade IV) and left facial paralysis at age 69 (House–Brackmann grade III), leading to right lower lip twitching and corner drooping. Hypertonicity of the DAO and DLI on the right side was identified as the cause. A myectomy of the DAO and DLI, along with PBFP flap placement, was planned. The facial flap was elevated, and the DAO and DLI were identified and excised, creating defects measuring 40 × 20 mm ( Fig. 3A ). A PBFP flap measuring 40 × 25 mm, including the capsule, was elevated and placed into the depression ( Fig. 3B–D ). On the left side, the lower lip had deviated upward due to weakened DLI force caused by complete paralysis. To address this, the double fascia graft technique 11 was utilized to pull the lower lip downward, thereby achieving symmetry of the lower lip. One year postoperatively, no depression was observed at the myectomy site. An improvement in ∠abx was achieved from 13 degrees to 5 degrees pre- and postoperatively, resulting in a natural, balanced smile ( Fig. 4A–D ).

These patients were independently evaluated by two board-certified plastic surgeons. Written informed consent was obtained from both patients for publication of this case report and accompanying images and video.

## Discussion

Botulinum toxin injections and selective neurectomies have been used to treat synkinesis and facial contractures associated with facial paralysis. The former is non-invasive but requires lifelong maintenance, 7 while the latter has a risk of recurrence due to postoperative reinnervation, even when performed by experienced surgeons, and often necessitates further chemodenervation. 12 Selective myectomy, initially developed for blepharospasm, has also been used to treat facial nerve palsy; however, it frequently results in postoperative depressed deformities. 1 13 Fat injection is a common treatment for such deformities following orbicularis oculi excision 7 13 ; however, its effectiveness is limited by low survival rates and difficulty in maintaining proper positioning of the injected fat. 14 Procedures using dermal fat grafts or SMAS pieces have been reported; however, they pose disadvantages such as donor site sacrifice, low survival rates, induration, scar contracture, and infection. 5 Given the proximity of the buccal fat pad to the myectomy site in this study, it was proposed that elevating it as a pedicle flap to augment the depressed area with vascularized tissue could mitigate these risks.

Clinically, the PBFP flap has been widely used to reconstruct defects in the cheek, oropharynx, palate, and sinus fistula, 8 9 with a high success rate due to its rich blood supply from the maxillary artery (buccal and deep temporal branches), superficial temporal artery (transverse facial branch), and facial artery (small branches). 8 These vessels also possess loose, tortuous structures that can effectively buffer against slight compression or stretching. 8 10 Anatomically, it is a random-pattern flap and has been reported to cover defects of up to 50 × 50 mm, 15 making it suitable even for combined excisions of the DAO and DLI. Baumann and Ewers have reported that the buccal fat pad maintains a constant size among different individuals, regardless of body weight and fat distribution, making it a reliable option even in patients with cachexia who have minimal subcutaneous fat. 16 Thus, it can be universally applied across diverse patient profiles.

Myectomy of the lower-lip depressor muscles is frequently used to treat facial contractures and marginal mandibular paralysis. However, this approach carries a high recurrence rate due to indistinct muscle boundaries, often necessitating resection of a slightly larger muscle width than is visually apparent. 17 Curtin et al. suggest resecting 2.5 to 3 cm of muscle length to minimize recurrence. 18 Sufficient-volume myectomy is crucial to prevent recurrence. Furthermore, Volk et al. reported that the thickness of the DAO and DLI increases by more than 20% in cases of chronic facial nerve paralysis associated with synkinesis and hypertonicity, 19 highlighting the possibility of postoperative depression deformity. Consequently, the placement of a PBFP flap is important for maintaining facial symmetry and preventing postoperative perioral depression.

Another possible cause of recurrence is that the reconnection of resected muscle fibers through scarring could result in the gradual recovery of muscle function. 20 PBFP flaps are also expected to serve as a spacer to hamper muscle reconnection.

This study has some limitations. A small sample size of only two patients was used, which restricts the generalizability of the results. Further research with a larger cohort is needed to validate the efficacy and applicability of this technique.

Additionally, the 1-year follow-up period was relatively short, preventing a full assessment of long-term results. Continued monitoring is necessary to identify potential delayed depressive deformities or scar contractures.

Overall, myectomy is an effective technique for managing partial facial contractures or synkinesis. Combining this approach with a buccal fat pad flap is a useful method for simultaneously correcting depression deformities resulting from myectomy.
